# “Could you sit down please?” A qualitative analysis of employees’ experiences of standing in normally-seated workplace meetings

**DOI:** 10.1371/journal.pone.0198483

**Published:** 2018-06-26

**Authors:** Louise Mansfield, Jennifer Hall, Lee Smith, Molly Rasch, Emily Reeves, Stephen Dewitt, Benjamin Gardner

**Affiliations:** 1 Department of Life Sciences, College of Health and Life Sciences, Brunel University, Uxbridge, United Kingdom; 2 Department of Psychology, Institute of Psychiatry, Psychology and Neuroscience, King’s College London, London, United Kingdom; 3 The Cambridge Centre for Sport and Exercise Sciences, Department of Life Sciences, Anglia Ruskin University, Cambridge, United Kingdom; 4 Department of Epidemiology and Public Health, University College London, London, United Kingdom; Swinburne University of Technology, Baker Heart and Diabetes Institute, Melbourne, AUSTRALIA

## Abstract

Office workers spend most of their working day sitting, and prolonged sitting has been associated with increased risk of poor health. Standing in meetings has been proposed as a strategy by which to reduce workplace sitting but little is known about the standing experience. This study documented workers’ experiences of standing in normally seated meetings. Twenty-five participants (18+ years), recruited from three UK universities, volunteered to stand in 3 separate, seated meetings that they were already scheduled to attend. They were instructed to stand when and for however long they deemed appropriate, and gave semi-structured interviews after each meeting. Verbatim transcripts were analysed using Framework Analysis. Four themes, central to the experience of standing in meetings, were extracted: physical challenges to standing; implications of standing for meeting engagement; standing as norm violation; and standing as appropriation of power. Participants typically experienced some physical discomfort from prolonged standing, apparently due to choosing to stand for as long as possible, and noted practical difficulties of fully engaging in meetings while standing. Many participants experienced marked psychological discomfort due to concern at being seen to be violating a strong perceived sitting norm. While standing when leading the meeting was felt to confer a sense of power and control, when not leading the meeting participants felt uncomfortable at being misperceived to be challenging the authority of other attendees. These findings reveal important barriers to standing in normally-seated meetings, and suggest strategies for acclimatising to standing during meetings. Physical discomfort might be offset by building standing time slowly and incorporating more sit-stand transitions. Psychological discomfort may be lessened by notifying other attendees about intentions to stand. Organisational buy-in to promotional strategies for standing may be required to dispel perceptions of sitting norms, and to progress a wider workplace health and wellbeing agenda.

## Introduction

Half of UK workers are office based [1]. Office workers spend approximately two-thirds of the working day in seated tasks [2] and are estimated to accumulate around 10.5h of sitting per waking day [3]. Growing evidence has associated prolonged sitting, characterised by very low energy expenditure (1.5 metabolic equivalents or less) [4], with increased disease and premature mortality risk [5–8]. Sedentary office work is an urgent public health issue. There is some evidence to suggest that some of the deleterious health effects of sitting time can be offset by engaging in moderate physical activity for at least one hour daily [9]. However, based on self-report data–which typically overestimate true activity levels [10]–a third of the UK population fails to meet the more modest target of 150 minutes of moderate activity per week [11]. It may be unrealistic to expect office workers who are highly physically inactive and highly sedentary to adopt daily bouts of activity lasting an hour or more [12]. Displacing sitting with standing may offer a more feasible sitting-reduction strategy.

Standing at work is often proposed as a way of reducing sitting [13–16]. Standing has been linked to lowered mortality rates [17], likely due at least in part to greater energy expenditure, reduced glucose variability and oxidative stress [11, 18, 19]. While there are no government guidelines offering targets for standing and sitting time in the UK, a recent expert-consensus document recommended that office workers accumulate 2-4h of standing and light activity daily, and take regular breaks from prolonged sitting [16]. Achieving this target will require developing strategies to displace sitting with standing. While the most commonly-evaluated strategy has been to restructure the environment to facilitate desk work while standing or moving [20–24], another commonly-proposed strategy is to promote standing in normally-seated meetings [25–26].

There are multiple routes through which standing in meetings might be widely adopted within the workplace. At the organisational and environmental levels, managers could implement initiatives that explicitly support standing meetings among employees, such as introducing standing-permissive meeting room furniture, or enshrining standing meetings into workplace practices and procedures [27]. Indeed, standing meetings are commonplace in the software development sector, where they are used for short daily team briefings [28]. At the individual level, employees might drive organisational change by voluntarily choosing to stand in meetings, with the aim of normalising standing in typically-seated contexts. Such a strategy, however, depends on the acceptability of standing in meetings to those who attempt it.

Interview studies suggest that office workers believe standing in meetings to be an acceptable sitting-reduction strategy *in principle* [29,30], but few studies have documented *lived experiences* of attempting to stand in normally-seated meetings. The scant available research into standing meetings to date has focused on responses to the topic matter and meeting frequency [28], not the standing experience itself. Initial experiences of novel behaviours are important determinants of maintenance [31, 32]; the sustainability of standing in meetings where all others are seated will depend not only on prior expectations, but also whether experiences match expectations [32, 33]. Employees are unlikely to continue to stand in seated meetings where they find the experience intolerable or unsuitable [33].

This paper reports the first study to our knowledge to examine employees’ experiences of standing in seated meetings using qualitative data. Understanding how employees experience standing in meetings, and the organisational, social and psychological structures that surround such experiences, will help to inform development and implementation of worksite standing interventions that go beyond the provision of sit-stand workstations. Our study focused on university workers. Universities are complex organisational structures offering diverse office environments, with workers from across the socioeconomic spectrum, so university workers’ experiences may potentially be relevant to many other office workers and settings. Our research question was: “how do office workers experience standing in normally-seated meetings?”

## Methods

### Participants

Participants were recruited from three UK-based universities, between January-April 2016, using convenience sampling methods. Recruitment was conducted using print and online advertisements through university communication channels (internal email, poster, staff newsletter, Twitter). Participants were offered a £50 (~US$65) voucher for taking part. Potential participants returned by email an expression of interest form, and self-reported their demographics (work/job role, gender, age, disability, ethnicity, income; Table 1) and eligibility, via items assessing the following criteria. Eligible participants were: desk-based employees; aged 18+ years with no intention to leave the organisation before July 2016; able to identify three meetings, differing in size, with potential for standing; and willing to be observed at meetings and interviewed. Those who had engaged in standing up in meetings in the past 3 months, used a sit-stand desk in the past 3 months, were full time students, or were unable to stand, were excluded. Those meeting eligibility criteria took part in a face-to-face, telephone or email inception meeting to clarify study requirements and agree meeting dates.

**Table 1:** Participant characteristics.

*Pseudonym*	*Workplace*	*Gender*	*Age*	*Ethnicity*	*Highest Qualification*	*Job Role*	*Monthly income (£k/month)*	*First meeting size*^a^	*Second meeting size*^a^	*Third meeting size*^a^
James	A	Male	16–24	White British or White other	Degree or higher	International Activities	1.5–2.4	S	M	L
Leila	A	Female	45–49	White British or White other	Degree or higher	Administration	1.5–2.4	L	M	S
Rosie	A	Female	30–34	White British or White other	Degree or higher	Academic	3.9+	L	S	M
Anusha	A	Female	25–29	Mixed	Degree or higher	International Activities	1.5–2.4	S	L	M
Sophie	A	Female	25–29	White British or White other	Degree or higher	Administration	1.5–2.4	S	M	L
Jane	A	Female	40–44	White British or White other	Degree or higher	Administration	2.4–3.9	S	M	L
Philip	A	Male	30–34	White British or White other	Degree or higher	Administration	2.4–3.9	S	L	M
Charlie	B	Other	25–29	*(Prefer not to say)*	Degree or higher	Administration	1.5–2.4	S	M	L
Joan	B	Female	40–44	Asian/Asian British	Degree or higher	Library & information services	3.9+	M	S	L
Ben	B	Male	50–54	Mixed	Degree or higher	Academic	1.5–2.4	M	L	S
Karim	B	Male	35–39	Black/Black British/Black other	Degree or higher	Academic	2.4–3.9	S	L	M
Tom	B	Male	35–39	White British or White other	Degree or higher	Academic	3.9+	L	S	M
Amelia	B	Female	25–29	White British or White other	Degree or higher	Academic	Prefer not to say	S	M	L
Graham	B	Male	35–39	White British or White other	Degree or higher	Administration	3.9+	M	S	L
Jas	B	Female	25–29	White British or White other	Degree or higher	Academic	2.4–3.9	S	M	L
Nadia	B	Female	16–24	White British or White other	Degree or higher	Academic	0.87–1.5	M	L	S
Eisha	B	Female	35–39	Black/Black British/Black other	Degree or higher	Human Resources	2.4–3.9	S	M	L
Brianna	C	Female	25–29	Mixed	Degree or higher	Administration	1.5–2.4	S	M	L
Anne	C	Female	25–29	White British or White other	Technical or professional	Public relations	2.4–3.9	M	S	L
Alisha	C	Female	35–39	White British or White other	Degree or higher	Administration	2.4–3.9	S	M	L
Jess	C	Female	40–44	White British or White other	Degree or higher	Administration	2.4–3.9	L	S	M
Angela	C	Female	25–29	Mixed	Degree or higher	Other	0.87–1.5	L	S	M
Zhen	C	Female	35–39	Asian/Asian British	Degree or higher	Administration	2.4–3.9	M	L	S
Rachel	C	Female	45–49	White British or White other	Technical or professional	Administration	2.4–3.9	S	M	L
Else	C	Female	30–34	Other	Degree or higher	Senior Management	1.5–2.4	M	S	L

^*a*^ Meeting size: S = Small (3–10 attendees), M = Medium (11–19 attendees), L = Large (20+ attendees).

Due to deadlines imposed by the broader project within which this study was located, a six-month window was set for study planning, data collection and analysis, which included three months for recruitment. Twenty-seven participants were recruited during this time, of whom two (from Workplace A) dropped out, citing insufficient time for participation, after the first meeting. The final sample thus comprised 25 participants (7 from Workplace A, 10 from Workplace B, 8 from Workplace C). Of these, six (24%) were male, 18 (72%) female, and one (4%) self-declared as ‘other’. The modal age category was 25-29y. Twenty-three (92%) participants had degree level or higher qualifications, and two (8%) had technical or professional qualifications.

### Procedure and interview schedule

As part of the pre-study information sheet, participants were given minimal information on the potential health benefits of standing (“public health researchers have suggested that office-based workers should stand up in meetings, to promote health”), and were told that “the study aims to explore what it is like for office-based employees to stand in meetings”. Participants were asked to select three group workplace meetings that they had been due to attend irrespective of study participation, and which differed in size (small: 3–10 attendees; medium: 11–19; large: 20+), such that each participant attended one meeting of each size (i.e., three meetings in total). We determined three meetings to be sufficiently conducive to variation in experiences for each participant, and feasible within the study period.

We instructed participants to stand whenever they felt they wanted and for durations decided by them. No further instruction was given. Except for five meetings (covering three participants) to which access was denied to non-invitees, a researcher attended each meeting to observe participants’ standing behaviour, others’ responses, room layout and number of attendees, though these field notes were not deemed sufficiently rich for analysis. A semi-structured interview (face-to-face or telephone) was conducted as soon as possible (and no longer than 48 hours) after each meeting (i.e. 3 interviews per participant), to gather reflections on experiences of standing. One participant (Amelia) attended her third meeting immediately following the second meeting, and so completed one interview addressing both meetings. Interviews took place within the meeting venue or another setting within the participant’s workplace, according to participant preference. Nobody else was present during interviews.

An interview schedule was developed to cover the fundamental determinants of standing (capability, opportunity, motivation [34]), and piloted among three office-based colleagues of the researchers. In all interviews, questions focused on affective reactions to standing; others’ reactions; standing location, timing and duration; occupational identity and status; workplace culture and norms; and acceptability and feasibility of standing in meetings. In the third interview, views towards workplace standing interventions were also sought. Each interview was conducted by one of three female researchers, one of whom (JH) was a Social Sciences doctoral student with previous experience of interviewing in the workplace sedentary behaviour research domain, and two (MR, ER) were Health Psychology Masters students trained in interviewing by the senior authors (LM, BG). Each participant was interviewed by the same interviewer on three occasions. The only interviewer-participant contact prior to the first interview was via email or telephone, for the purposes of organising the first interview. Participants were told, prior to the interview, that the interviewer was conducting the study as part of a funded research project (JH), or as coursework (MR, ER). No other interviewer characteristics were shared with participants prior to the first interview. Interview duration ranged from 8-32mins (mean 20mins).

The study was approved by the Research Ethics Committee of each institution (LRU-15/16-2533; 4385/002; 1875-LR-Jan/2016-1200). All participants provided fully informed written consent to participate.

### Analysis

Interviews were digitally audio-recorded and transcribed verbatim, with transcript quality and completeness verified by the researchers. We offered to return transcripts, and our ongoing or completed analyses, to participants for comment, correction, or any other purpose, but none expressed a wish to receive them.

Interview transcripts and analysis were managed via NVivo 10 software. A phenomenological methodological orientation was adopted, as this allows for description (rather than quantification or explanation) of participants’ experiences, and exploration of the subjective meanings that they ascribe to elements of these experiences. Data were analysed using Framework Analysis, which allows inductive co-creation between multiple researchers of an initial coding framework which guides subsequent analysis, and which is developed and iteratively refined as coding progresses and new insights emerge [35–37]. Repeated and independent reading of a sample of interview data from one institution was undertaken by two researchers (JH, LM), to develop a preliminary coding framework. Next, selected interview transcripts (n = 9/25) were read and re-read by four other researchers (MR, LS, ER, BG), who refined the coding framework to ensure its accuracy and relevance. Discrepancies were resolved by discussion, and resultant codes and themes verified by all researchers. The coding framework was used (by JH, LM, and BG) to analyse the remaining data. Given that data collection was constrained by the study timeline, data saturation was not discussed prior to analysis.

Quotes are provided below as evidence of the validity of our analysis [38]. All participants were assigned pseudonyms. Punctuation was added to unambiguous quotes and where necessary, words added in parentheses to clarify intended meaning.

## Results

Four themes were derived from the data: physical challenges to standing; implications of standing for meeting engagement; standing as norm violation; and standing as appropriation of power. The first theme details experiences of physical discomfort and of attempts to negotiate the physical environment to permit standing. The second theme reports different ways in which standing impacted on participants’ involvement in meetings. The third and fourth themes describe discrete psychological challenges involved in negotiating the organisational and inter-person context that frames workplace meetings.

### Theme 1: Physical challenges to standing

Some participants were aware of the detrimental impact of sitting and expected that standing in meetings would confer health benefits (“*you just perceive being standing up*, *it’s just better for your health and it just makes you feel better overall”*; James, Workplace A). Yet, the physical experience of standing in meetings rarely mapped onto participants’ expectations. Some reported unexpected and unaccustomed discomfort from physical sensations in the muscles of the feet, legs, back and shoulders, while others anticipated but did not experience physical discomfort:

*I just thought, oh your back will ache or legs ache or something, but actually it wasn’t borne out in reality*.

(Tom, Workplace B).

The physical impact appeared partly due to the time spent standing, which was self-determined by participants. Some expected to be able to stand for the entirety of lengthy meetings, but on attempting to do so realised this was not possible, due to physical discomfort:

*After about twenty-five minutes … [I was] thinking oh my back is killing me! There’s the realisation that, oh I can’t stand for very long*!

(Joan, Workplace B)

Aspects of the physical environment–furniture design, spatial configuration, and the numbers and positions of attendees–also presented barriers and challenges to standing. Participants’ descriptions of their meeting room environments suggested that there were many chairs, but no standing areas, nor desks and tables to support standing (a “*lack of furniture for standing”*; Angela, Workplace C). The physical environment was felt to elicit sitting (*“the [physical] environment … kind of shapes social expectations [about standing] … we sit so much”*; Angela, Workplace C), which in turn reinforced perceptions that standing in meetings was neither acceptable nor feasible:

*Today, with the chairs all round, already set up, it makes it more of a barrier to actually saying I’m going to stand*… *it’s so easy just to go, oh I’m going to sit down*

(Tom, Workplace B)

A lack of perceived environmental support for recording information when standing posed practical difficulties for several participants, making engaging in the meeting non-ergonomic and potentially physically uncomfortable:

*Bending down and trying to take notes, it didn’t feel natural*. *It was just a question of the desk or table not being at a certain height really*

(Charlie, Workplace B)

Suggestions were made for overcoming barriers presented by ill-suited physical environments, including furniture adaptation, or using accessories to permit usual meeting engagement while standing; for example, using a tablet instead of pen and paper for note-taking.

### Theme 2: Implications of standing for meeting engagement

Many participants reported that standing affected their engagement in the meeting. Some found that physical discomfort from standing motivated them to increase engagement to minimise meeting length:

*Because I was stood … [I was] kind of more, more ‘let’s get on with it, let’s get to the point’. […] Because it’s not so relaxed as being sat back in a chair … the perception is the meeting is going on for longer, or more frustration if there’s no action being taken. […] It’s more efficient*.

(Tom, Workplace B)

Others felt that shifting from a relaxed, seated position to standing sustained focus on the meeting, because standing prevented them from “*switching off*” (James, Workplace A) as they would while sitting.

Many reported, at least in the first of the three meetings in which they stood, unanticipated feelings of psychological discomfort from standing while all others were sitting, together with a heightened awareness of the self, others, and the interpersonal context. Participants variously felt *“disconcerted”* (Joan, Workplace B), *“awkward”* (Brianna, Workplace C), or *“stupid”* (Charlie, Workplace B), possibly due to being more visible than others. Some reported that enhanced visibility made them feel more accountable to others:

*I tend to drift out or find them a bit boring. […] Because I was both at the front of the room and standing, I felt much more like I had to, even if I wasn’t engaged, look like I was more engaged, which then made me more engaged. So I actually listened to the whole thing! […] If I started drifting out … I’d then get really self-conscious and think, oh God, what if somebody saw me with my glazed-over eyes or something like that*!

(Anne, Workplace C)

For others however, self-conscious thoughts were a distraction from the meeting:

*When I did sit down, I was like (sighs). More relaxed. I could just focus on the meeting, not focus on my standing*.

(Brianna, Workplace C)

Some participants sought to minimise psychological discomfort by standing in a position within the room that they perceived to be less visible, so avoiding obstructing others:

*I positioned myself right towards the back in the corner… it was OK to stand because I wasn’t making a nuisance of myself to anybody, I wasn’t in any body’s way*.

(Anusha, Workplace A)

However, physically removing themselves from others was experienced by some as isolating, and potentially limited involvement in interactions within the meeting:

*Standing up made me … [feel] like I wasn’t part of the group. … [Once I sat down] I felt like I was then part of the meeting. And it felt more like we were a team group coming to some decisions and stuff, because we were all on the same eye level*.

(Alisha, Workplace C)

*I got missed out on the signing register. I didn’t draw attention to myself because I wanted someone to notice me and give it to me, and they didn’t*.

(Selma, Workplace C)

Some felt that other attendees were preoccupied with their standing, which might have compromised the engagement of others, and limited overall satisfaction with the conduct of meetings:

*They were kind of looking at me instead of looking at the director… I think I just felt like I was being a distraction for them [others in the meeting], I felt like I was taking away from the meeting*.

(Brianna, Workplace C)

Some were concerned that standing could be interpreted by others as unwillingness to fully engage in the meeting, and indeed, a minority of participants reported being asked by others to sit for this reason:

*[In a meeting] you expect someone to sit down and then [if] they don’t you think, are you not staying or do you not really want to have the meeting*?

(Alisha, Workplace C)

*She [the chair of the meeting] goes: “it’s really distracting with you standing up. It feels like you’re getting ready to go, could you sit please?” So then I sat*.

(Brianna, Workplace C)

Many felt it particularly inappropriate not to sit in formal meetings, or those addressing sensitive topics, as it risked diminishing the seriousness of the meeting:

*Knowing that [the meeting topic] is actually maybe quite confidential or sensitive, I don’t want to be standing up. I need to be sitting down*.

(Else, Workplace C)

### Theme 3: Standing as norm violation

Many participants found that standing in all-seated meetings made salient the prevailing implicit norm of sitting in meetings, and their deviation from this behavioural standard.

*As soon as it’s called a meeting, it formalises everything. There are just certain social expectations and standing is not one of them. […] I felt like I was breaking the rule. […] As with any social norm, as soon as you’re in a position where you might be going against it, you suddenly feel the weight of society’s expectations on you*.

(Angela, Workplace C)

Participants worried that standing would be seen as a deliberate attempt to challenge the sitting norm, and that they would be perceived by other attendees as “*an attention seeker*” (Ben, Workplace B), willfully detracting from the business of the meeting. Some participants were concerned that being seen to be violating the norm could potentially detrimentally impact other attendees, and so the progression and outcomes of the meeting:

*I would worry that I was making them [other attendees] uncomfortable and worry that they wouldn’t be able to have the meeting that they wanted, and that they wouldn’t get out of it what they wanted or not be able to talk as freely as they would normally*.

(Alisha, Workplace C)

Characteristics of the meeting context–the perceived formality, purpose, type, length, and size of the meeting, and relationships between attendees–shaped the standing experience for many, often affecting the extent to which participants felt compelled to conform to the sitting norm. Meeting contexts that made participants’ contravention of the sitting norm more prominent were most aversively experienced. For example, meetings characterised by frequent interaction between attendees, larger meetings, and those where other attendees were unknown to the stander, were often cited as challenging. Many participants sought to deflect unwanted attention and avoid misconceptions from others by forewarning other attendees of their intention to stand, or seeking explicit permission from the meeting leader, in advance or at the outset of the meeting. Most felt that they had to explain their decision to stand to others, and while many truthfully cited involvement in our study, some felt that this provided insufficient justification, instead feigning ill-health to claim exemption from the sitting norm:

*I lied and told them I had a health reason for needing to stand*. […] *It’s just one of those things that*, *unless you have a good enough excuse to stand*, *they’re going to assume that you’re just being difficult*.

(Anusha, Workplace A)

Several participants recounted episodes in which their decision to stand was misinterpreted by others as reflecting a lack of opportunity to sit, which in turn was felt to obligate the participant to sit when such an opportunity was provided:

*I probably stood for about a minute … and then someone else looked at me and they were gesturing that they’d saved me a seat! […] I felt super awkward and sat down*.

(Angela, Workplace C)

### Theme 4: Standing as appropriation of power

Psychological discomfort appeared to arise not only from being seen to violate sitting norms, but also because standing was felt to affect the power dynamics of the meeting. Standing in an all-seated meeting was felt to symbolize status and authority within the meeting:

*You tend to think the more authoritative person, or the person that’s going to lead the conversation, might be the one that’s a bit higher*.

(Brianna, Workplace C)

Indeed, many reported that standing made them feel empowered:

*I probably addressed everyone and raised my voice a little, projected it a bit more than I might do … if I was seated. […] [Standing is] a much more confidence-boosting posture*.

(Joan, Workplace B)

Where the participant was hosting the meeting, the additional power conferred by standing was deemed useful for denoting and exercising leadership (“*I was the lead … so it seemed natural that I had that authoritative position*”; Anusha, Workplace A). Where participants were not leading the meeting, however, they worried that standing would be misconstrued as a tacit attempt to appropriate power by challenging the authority of the meeting leader, or other, more senior attendees:

*If everyone’s sat down there and you’re up there*, *there’s*, *you know*, *almost a visual representation of a hierarchy in a weird way*.

(Joan, Workplace B)

*I stood up while [my manager] sat down. I felt uncomfortable because I felt like I was telling her what to do, like I was like a teacher and she was a student because I was standing over her. […] I think she probably felt equally as uncomfortable*.

(Angela, Workplace C)

Meetings featuring presentations by one attendee to the group were typically less problematic, as were those in which senior attendees explicitly supported standing, because participants felt that there was little risk of standing being interpreted as an attempt to assert power in such situations:

*As a junior member in a meeting, I wouldn’t really be willing to stand up and say, well I’m standing up because I want to stand up. [But] I’d be more willing to, if … someone more senior said I’m standing up, I’d say, great, I’m going to join you*.

(Amelia, Workplace B)

Meetings held in familiar social or physical settings, especially locations over which the participant felt they already had ownership and authority (e.g. the participant’s office), were also less psychologically uncomfortable:

*Just the familiarity of the people in the team now, my relationship with the people in the team makes it easy to stand, I’m comfortable, you know if anyone made, no one’s made really any negative comments, but even if anyone did, I’d be comfortable just being like, well this is what I want to do*.

(James, Workplace A)

## Discussion

Standing in meetings is often proposed as a strategy by which to reduce workplace sitting [25, 29, 39, 40], but no study to our knowledge has yet documented how people experience standing in meetings in which all other attendees sit. Interviews with volunteers who stood in planned workplace meetings revealed barriers that workers can expect to face if deciding to stand where others are sitting, and potential facilitators. For many, the experience of standing in meetings was uncomfortable in some way. Some participants experienced physical discomfort from standing for self-imposed lengthy periods and spoke of practical challenges posed by the lack of suitability of meeting room furniture to standing. Perhaps moreover, participants felt psychologically uncomfortable about standing. This apparently arose from concerns about being seen to violate a compelling social norm favouring sitting, or being seen to be challenging the authority of other attendees by standing while others sat. Many participants chose to reduce the potential impact of their standing on others by removing themselves to the edges of the meeting room, though this risked limiting their involvement in the meeting. For meeting hosts, standing was often found to confer greater power, and enhance confidence. Our findings provide a much-needed illustration of the broader interpersonal and organizational contexts that frame workplace behaviour, and the difficulties these pose for standing in meetings. Promoting standing in normally-seated meetings requires that office-based organisations and workers anticipate these challenges.

It is important to mitigate potentially aversive consequences of standing in meetings; while office workers generally appear willing to try standing in meetings [39, 40], they are unlikely to continue to stand if initial attempts fail to attain expected positive outcomes or yield predominantly negative outcomes [33]. Our findings revealed several such negative outcomes, many of which participants did not foresee. Many reported physical discomfort, sometimes identified as pain, which appears to have been due to prolonged standing; many felt that they had to stand for the entirety of meetings, though we did not instruct them to do so. This likely reflects a misplaced belief among the public that the health risks of sitting can only be offset by prolonged standing [41]. Yet, standing still for long periods can also harm health [42–44]. A recent expert consensus statement on workplace sitting and standing [16] advises that “prolonged, static standing postures be avoided” (p1360), and that sitting be replaced by frequent sit-stand transitions, standing, and light physical activity. While physical activity is unlikely to be feasible in meetings, workers in lengthy meetings could realistically be encouraged to build standing time gradually, stand only for as long as is comfortable, and regularly alternate between sitting and standing.

Participants also described psychological discomfort resulting from ‘standing out’ from others in the meeting. This echoes previous research showing that people report feeling ‘weird’ or self-conscious from standing in normally-seated workplace contexts [25]. For many, such discomfort arose from knowingly violating a strong perceived social norm exerting pressure to sit and not stand. These findings support the centrality of social norms as a predictor of action, in potential competition with one’s own attitudes, such that people sometimes act in a counter-attitudinal way to conform to social pressures [45, 46]. Indeed, some participants reported that, despite wanting to stand, they aborted their standing attempts early, in response to implicit or explicit pressure from others to sit. Experiences of standing appeared to vary in accordance with the extent and visibility of perceived norm violation. In meetings in which standing seemed to more strongly contravene the implicit sitting norm–such as larger meetings characterized by discussion among attendees–standing produced stronger feelings of awkwardness. Particularly where highly emotive topics–such as job losses–were under consideration, many participants felt that their decision to stand could be misinterpreted as a challenge to the seriousness of the meeting, or the authority of attendees.

Standing also elicited psychological discomfort for some because they feared that their standing would be interpreted as an assertion of power and authority over other attendees. Indeed, for many animals, moving from sitting to standing can be a sign of dominance and aggression [47], and previous research has documented concerns among office workers about the potential for standing to be misconstrued as aggressive or threatening [40]. Concern about such misperceptions was an especially powerful barrier to standing in meetings with more senior colleagues. In meetings in which participants felt that standing could not perceived to be an infraction–such as when standing to present information, or when participants considered they had a legitimate reason to stand–standing did not appear to elicit strong feelings. Interestingly, however, many felt that current health problems precluding prolonged sitting offered the only legitimate rationale for standing. This testifies to the perceived strength of the sitting norm, and of being seen to respect established hierarchical relations within the meeting; some believed that standing for the sake of health promotion (rather than management of ill-health), or to honour commitments to participate in our study, did not constitute sufficient reasons to disrupt norms or be seen to challenge the authority of others. Thus, where meetings were held in settings over which the participant perceived a sense of ownership and authority–such as where the participant was leading the meeting–standing was seen as less of a contravention of norms, and no threat to established power dynamic, so did not evoke psychological discomfort.

Together, our findings suggest several potentially fruitful strategies for overcoming norm barriers. First, as many of our participants found, notifying others–especially meeting leaders–of intentions to stand, or relocating to a less visible position in the room, can alleviate perceived social pressure to sit. Relocation can, however, yield mixed consequences. In meetings characterized by interaction among attendees, some participants felt that standing physically and psychologically distanced them from collaborative discussions. Others reported that standing could prompt more efficient meetings, due to the desire to minimize anticipated physical discomfort from prolonged standing. Indeed, previous research suggests that standing meetings tend to be shorter in duration than seated meetings, with no impact on the quality of meeting outcomes [48]. Second, and more broadly, office-based organisations should explicitly promote standing in meetings, to counter the perceived sitting norm, and thus empower those who wish to stand without fear of infringing social expectations. Meeting hosts should also encourage standing in meetings; participants often felt more confident standing when they had secured prior approval from those leading the meeting. Hosts might, for example, suggest that attendees must stand when speaking in contribution to a group discussion, a strategy shown to be acceptable in principle in a study of employees in Belgium [29]. Managers may be encouraged to support standing in meetings through emphasizing potential benefits to productivity and staff time arising from shorter, more efficient meetings [25, 29, 48]. Third, there is an urgent need to promote standing for health promotion purposes in the workplace, by developing messages that frame standing as a legitimate strategy for sitting less. Given also the pervasive culture of sitting cited by our participants, organisational buy-in, involving creating and making salient and explicit a standing-permissive culture, will be central to the effectiveness of promotional strategies for standing in meetings [27, 49]. Organisations can also facilitate standing in meetings by providing standing-appropriate infrastructure, such as meeting spaces with high tables and stools [25]. While many participants adapted to standing in sitting-conducive environments by using accessories (e.g. using tablets to take notes), restructuring of the physical environment has been shown to be an effective upstream method for reducing sitting among workers [20, 50, 51].

Study limitations must be acknowledged. We did not assess participants’ prior sitting time or the frequency with which they attended meetings, nor were measures taken of the length of time for which participants stood, all of which may have influenced responses to standing. Moreover, as we showed, the experience of standing in meetings is influenced by the perceived responses of other attendees, but we did not collect data from meeting attendees other than the standing participant. Standing may create psychological discomfort among non-standing attendees, in turn feeding negative perceptions of the stander. While we have focused our practical recommendations on how to minimize feelings of discomfort among standers, effectively promoting standing in meetings may perhaps also require understanding and assuaging potentially negative experiences of non-standing attendees.

We sought to explore real-world experiences of standing in meetings, but study procedures may have influenced such experiences. Participants may at least in part have been incentivized to stand by the gift voucher incentive, or by the belief that participating in our study in this way would contribute to scientific knowledge. It is thus possible that their reasons for and experiences of standing among our sample may differ to those of employees who would stand in meetings in more naturalistic settings, thus questioning the representativeness of the experiences we documented. We sought to minimize our influence on the standing experience by giving minimal instructions to participants, asking only that they attempt to stand for a self-determined time. However, a lack of further instruction ironically appears to have had an important influence on experiences; the physical discomfort reported by many was apparently due to the misconception that participants should stand for as long as possible. Participants may have had more positive experiences had they, for example, been advised on how best to integrate standing into meetings, including setting realistic standing duration goals, and informing meeting hosts and other attendees in advance. Nonetheless, our methods have documented the potential importance of informing others of decisions to stand, and of the potential for people to misunderstand advice to stand *more* as a recommendation to stand for as long as possible [41]. We are confident that our findings offer valid insights of importance for informing future guidance for incorporating standing into meetings.

Our sample was small and, while university employees span a socioeconomically broad range, our participants were highly educated, which questions the generalizability of findings. However, participants were recruited from three office-based university organisations, and captured a diversity of meeting types, job roles and seniority. Moreover, our aim was not to identify a generalizable set of experiences, but rather to capture and explore a range of reflections on the experience of standing in meetings. Indeed, while previous research has suggested that office workers find the idea of standing in normally-seated meetings acceptable in principle, ours is the first study to document the rich complexity of the psychological, interpersonal and organisational contexts that frame the standing experience.

## Conclusions

Displacing sitting with standing at work requires an in-depth understanding of how to integrate standing into normally-seated work practices. While meetings offer but one workplace context in which sitting time might be reduced, our study demonstrates the complexity of this specific context, which should be acknowledged by future workplace sitting reduction initiatives. Specifically, we have highlighted some important physical, psychological and social barriers and facilitators that may determine whether someone feels sufficiently capable to break the mould and stand in normally-seated meetings. Office workers must acknowledge that standing in meetings will involve a period of acclimatisation to an unusual way of working. Many of our participants learned to adapt to standing over the course of the three meetings, and so reduced initial physical and psychological discomfort. Strategies that may enable office workers to sustainably adopt standing in meetings as a sitting-reduction strategy include building standing time gradually, and alternating between sitting and standing, to alleviate physical discomfort, and notifying attendees of intentions to stand, to avoid psychological discomfort from being seen to be challenging norms and social hierarchy. Office managers should seek to provide visible organizational support for standing, including the explicit promotion of the acceptability of standing in the workplace as a health promotion strategy, and provision of designated areas of standing-supportive furniture.

## Supporting information

S1 File Interview schedules. (DOCX)

S2 File Coding tree. (DOCX)

S3 File Completed COREQ checklist. (DOCX)
