# Supplementary material for: “Could you sit down please?” A qualitative analysis of employees’ experiences of standing in normally-seated workplace meetings
Source: PLoS One. 2018 Jun 26;13(6):e0198483. doi: 10.1371/journal.pone.0198483 (PMC6019091; doi:10.1371/journal.pone.0198483)
Supplement: S1 File — (DOCX) [file pone.0198483.s001.docx]

**Supporting information (S1): Interview schedules**

*Pre-interview checklist:*

- *Reminder of the aims of the project / interview – “we are interested in your experience of standing in meetings”*
- *Attain verbal consent to be interviewed from interviewee (consent form will have been completed prior to standing in meeting)*
- *Ask for consent to record the interview*
- *Ask if the interviewee has any questions for you before you start the interview*

**First interview: Topics / suggested questions**

- *Motivation to participate*
  - Why did you want to take part in this study?
- *Motivation to stand*
  - Why were you interested in standing in meetings initially?
  - Before this meeting, did you want to stand or are you sceptical about standing in meetings?
  - What do you understand the benefits of standing in meetings to be/ did you experience any benefits of standing in this meeting?
- *Expectations about standing*
  - What were your prior expectations about standing in meetings?
- *Experience of standing*
  - Describe your standing experience in this meeting
    - (PROMPTS: did it differ from expectations and in what way? At what point did you stand and why? Did you sit and why? Did you stand again and why? Did you tell people that you were going to stand or did you just do it? Did you explain why you were standing to anyone? How did standing make you feel?)
  - What factors contributed to your experience of standing?
- *Capability / Opportunity to stand*
  - Did anything restrict you from standing?
    - (PROMPTS e.g. needing to make notes, physical environment, other workplace factors listed above such as other participants and meeting size)
  - Did anything facilitate your standing?
  - Would anything make it easier for you to stand, or make you more willing to stand?
- *Workplace context for standing, norms and habits in meetings*
  - Did it seem ‘normal’ or was it a new experience for you?
  - How did others in the meeting react to you standing?
  - Do you stand in any other aspects of your job?
  - Is the workplace / meetings an appropriate context to stand?
  - Does (University) provide opportunities for you to reduce sitting / are they supportive of it?
  - Is your manager supportive?
- *Is there anything else you’d like to discuss?*

**Second interview: Topics / suggested questions**

- *Experience of standing*
  - Describe your standing experience in this meeting
    - (PROMPTS: did it differ from expectations and in what way? At what point did you stand and why? Did you sit and why? Did you stand again and why? Did you tell people that you were going to stand or did you just do it? Did you explain why you were standing to anyone? How did standing make you feel?)
- *Capability, Opportunity and Motivation to stand (COM-B)*
  - Before this meeting, did you want to stand? Why?
  - Did anything restrict you from standing?
    - (PROMPTS e.g. needing to make notes, physical environment, other workplace factors listed above such as other participants and meeting size)
  - Did anything facilitate your standing?
  - Would anything make it easier for you to stand, or make you more willing to stand?
- *Change in behaviour elsewhere*
  - Has standing in meetings affected your sitting, standing or physical activity outside of meetings?
    - (PROMPTS: Change in (sitting/standing) behaviour outside of meetings? Change in light/moderate/vigorous PA in the workplace or outside the workplace? Change in attitude towards sitting and standing? Change in awareness of own sitting and standing behaviours and patterns?
- *Is there anything else you’d like to discuss?*

**Third interview: Topics / suggested questions**

- *Experience of standing*
  - Describe your standing experience in this meeting
    - (PROMPTS: did it differ from expectations and in what way? At what point did you stand and why? Did you sit and why? Did you stand again and why? Did you tell people that you were going to stand or did you just do it? Did you explain why you were standing to anyone? How did standing make you feel?)
- *Capability, Opportunity and Motivation to stand (COM-B)*
  - Before this meeting, did you want to stand? Why?
  - Did anything restrict you from standing?
    - (PROMPTS e.g. needing to make notes, physical environment, other workplace factors listed above such as other participants and meeting size)
  - Did anything facilitate your standing?
  - Would anything make it easier for you to stand, or make you more willing to stand?
- *Change in behaviour elsewhere*
  - Has standing in meetings affected your sitting, standing or physical activity outside of meetings?
    - (PROMPTS: Change in (sitting/standing) behaviour outside of meetings? Change in light/moderate/vigorous PA in the workplace or outside the workplace? Change in attitude towards sitting and standing? Change in awareness of own sitting and standing behaviours and patterns?
- *Views on intervention ideas*
  - Do you think that standing in work meetings is possible / something that you would consider doing / continue to do?
  - What could workplaces to do to make it easier for people to stand in meetings / to promote standing in meetings?
- *Is there anything else you’d like to discuss?*
