# Supplementary material for: “Could you sit down please?” A qualitative analysis of employees’ experiences of standing in normally-seated workplace meetings
Source: PLoS One. 2018 Jun 26;13(6):e0198483. doi: 10.1371/journal.pone.0198483 (PMC6019091; doi:10.1371/journal.pone.0198483)
Supplement: S2 File — (DOCX) [file pone.0198483.s002.docx]

**Supporting Information (S2): Coding tree**

**Theme 1: Physical challenges to standing**

a. Physical discomfort

i. Anticipated but not experienced

ii. Not anticipated but experienced

iii. Potential causes

b. Physical environment

i. spatial arrangement – supports sitting norm

i. furniture design

a. lack of suitable furniture / need for more appropriate furniture

i. ill-suited to recording information

ii. ergonomically unsound

b. consequences of standing where furniture ill-suited

i. less able to record information

ii. greater physical discomfort

c. solution: (suggestions for overcoming barriers presented by inappropriate furniture)

i. furniture adaptation

ii. using accessories (e.g. clipboard, ipad) as workaround

**Theme 2: Implications of standing for meeting engagement**

1. Inability to maintain normal levels of engagement in meeting

i. Increased engagement / greater focus on meeting

a. physical discomfort

i. desire for shorter/more efficient meetings

b. more mentally alert and focused

1. Cognitive demand / distraction

i. Psychological discomfort arising from:

a. drawing undue/too much attention to oneself

i. Heightened awareness of self and others

a. preoccupation wth self-conscious thoughts

b. greater visibility

i. solution: people chose to stand in somewhere where they were

a. less visible

b. less likely to avoid obstructing others

ii. problem: removal as

a. isolating

b. limiting engagement

c. expected misconceptions of others

i. standing as distracting from meeting

ii. standing as sign of unwillingness to engage

a. solution: avoid standing in formal/serious meetings

**Theme 3: Standing as norm violation**

1. heightened awareness of sitting norm

i. awareness of norm violation

1. worry over others’ misconceptions
2. standing as attention-seeking

ii. standing as distracting for others

1. meeting-related factors affecting awareness/salience of norm violation

i. formality

ii. purpose

iii. type

iv. length

v. size

vi. relationship among attendees

1. solutions to norm violation:

i. informing/warning others of standing

ii. seeking permission from meeting host

iii. appeals to exemption

1. other sitting-related norms

i. perceived obligation to sit when offered seat

**Theme 4: Standing as appropriation of power**

1. standing as symbol of status and authority

i. standing as empowering for meeting hosts

1. standing as (mis)perceived challenge to others’ status

i. standing as assertion of authority

1. meeting settings less conducive to misperceptions of power appropriation

i. familiar settings

ii. informal settings
