# Supplementary material for: “Could you sit down please?” A qualitative analysis of employees’ experiences of standing in normally-seated workplace meetings
Source: PLoS One. 2018 Jun 26;13(6):e0198483. doi: 10.1371/journal.pone.0198483 (PMC6019091; doi:10.1371/journal.pone.0198483)
Supplement: S3 File — (DOCX) [file pone.0198483.s003.docx]

**Supporting Information (S3).** Completed COREQ checklist

| **Item** | **Guide questions/description** |  |  | **Relevant line(s)/page(s)/section** |
| --- | --- | --- | --- | --- |
| 1. Interviewer/facilitator | Which author/s conducted the interview or focus group? |  |  | Lines 148-152 |
| 2. Credentials | What were the researcher's credentials? *E.g. PhD, MD* |  |  | Lines 149-152 |
| 3. Occupation | What was their occupation at the time of the study? |  |  | Lines 149-152 |
| 4. Gender | Was the researcher male or female? |  |  | Line 149 |
| 5. Experience and training | What experience or training did the researcher have? |  |  | Lines 148-152 |
| 6. Relationship established | Was a relationship established prior to study commencement? |  |  | Lines 153-154 |
| 7. Participant knowledge of the interviewer | What did the participants know about the researcher? e*.g. personal goals, reasons for doing the research* |  |  | Lines 155-156 |
| 8. Interviewer characteristics | What characteristics were reported about the interviewer/facilitator? e.g. *Bias, assumptions, reasons and interests in the research topic* |  |  | Line 156-157 |
| 9. Methodological orientation and Theory | What methodological orientation was stated to underpin the study? *e.g. grounded theory, discourse analysis, ethnography, phenomenology, content analysis* |  |  | Lines 168-170 |
| 10. Sampling | How were participants selected? *e.g. purposive, convenience, consecutive, snowball* |  |  | Lines 96-108 |
| 11. Method of approach | How were participants approached? e*.g. face-to-face, telephone, mail, email* |  |  | Lines 97-99 |
| 12. Sample size | How many participants were in the study? |  |  | Lines 113-115 |
| 13. Non-participation | How many people refused to participate or dropped out? Reasons? |  |  | Lines 113-114 |
| 14. Setting of data collection | Where was the data collected? e*.g. home, clinic, workplace* |  |  | Lines 140-141 |
| 15. Presence of non-participants | Was anyone else present besides the participants and researchers? |  |  | Line 141-142 |
| 16. Description of sample | What are the important characteristics of the sample? *e.g. demographic data, date* |  |  | Table 1 (lines 109-110), Lines 115-119 |
| 17. Interview guide | Were questions, prompts, guides provided by the authors? Was it pilot tested? |  |  | Lines 143-145 |
| 18. Repeat interviews | Were repeat interviews carried out? If yes, how many? |  |  | Lines 137 |
| 19. Audio/visual recording | Did the research use audio or visual recording to collect the data? |  |  | Line 163 |
| 20. Field notes | Were field notes made during and/or after the interview or focus group? |  |  | Lines 132-135 |
| 21. Duration | What was the duration of the interviews or focus group? |  |  | Lines 157-158 |
| 22. Data saturation | Was data saturation discussed? |  |  | Lines 179-180 |
| 23. Transcripts returned | Were transcripts returned to participants for comment and/or correction? |  |  | Lines 164-166 |
| 24. Number of data coders | How many data coders coded the data? |  |  | Lines 173-179 |
| 25. Description of the coding tree | Did authors provide a description of the coding tree? |  |  | Supporting Information (S2) |
| 26. Derivation of themes | Were themes identified in advance or derived from the data? |  |  | Line 170-180 |
| 27. Software | What software, if applicable, was used to manage the data? |  |  | Lines 167-168 |
| 28. Participant checking | Did participants provide feedback on the findings? |  |  | Lines 165-166 |
| 29. Quotations presented | Were participant quotations presented to illustrate the themes / findings? Was each quotation identified? e*.g. participant number* |  |  | Line 181, Lines 194-362 |
| 30. Data and findings consistent | Was there consistency between the data presented and the findings? |  |  | Yes |
| 31. Clarity of major themes | Were major themes clearly presented in the findings? |  |  | Yes |
| 32. Clarity of minor themes | Is there a description of diverse cases or discussion of minor themes? |  |  | Yes (e.g. Lines 263-266) |
